# Adiposity measures and vitamin D concentrations in Northeast Germany and Denmark

**DOI:** 10.1186/s12986-015-0019-0

**Published:** 2015-06-10

**Authors:** A. Hannemann, B. Heinsbaek Thuesen, N. Friedrich, H. Völzke, A. Steveling, T. Ittermann, K. Hegenscheid, M. Nauck, A. Linneberg, H. Wallaschofski

**Affiliations:** Institute of Clinical Chemistry and Laboratory Medicine, University Medicine Greifswald, Greifswald, Germany; Research Centre for Prevention and Health, Glostrup Hospital, Glostrup, Denmark; Institute for Community Medicine, University Medicine Greifswald, Greifswald, Germany; Department of Medicine A, University Medicine Greifswald, Greifswald, Germany; Institute of Diagnostic Radiology and Neuroradiology, University Medicine Greifswald, Greifswald, Germany; Department of Clinical Experimental Research, Glostrup University Hospital, Glostrup, Denmark; Department of Clinical Medicine, Faculty of Health and Medical Sciences, University of Copenhagen, Copenhagen, Denmark

**Keywords:** Obesity, Vitamin D, Population-based, Abdominal adipose tissue, Body fat, Body mass index

## Abstract

**Background:**

Body mass index (BMI) and serum 25-hydroxy vitamin D3 (25OHD) concentrations are inversely related. As BMI contains only limited information regarding body fat distribution, we aimed to analyze the cross-sectional associations of abdominal visceral or subcutaneous adipose tissue, next to common adiposity measures, with the 25OHD concentration.

**Methods:**

Data were obtained from three cohorts of two large epidemiological studies in the northeast of Germany (Study of Health in Pomerania, SHIP-1 and SHIP-Trend), and in Denmark (Health2006). The study populations included adult men and women from the general population (*N* = 3072 SHIP-1, *N* = 803 SHIP-Trend, *N* = 3195 Health2006). Visceral and subcutaneous adipose tissue were quantified by magnetic resonance imagining (SHIP-Trend) or ultrasound (Health2006). Common adiposity measures, including BMI, waist circumference, waist-to-hip ratio, waist-to-height ratio, body surface area, and body fat percentage were determined by standardized methods in SHIP-1 and Health2006.

**Results:**

The average study participant was overweight (median BMI 27.4, 26.6, and 25.2 kg/m^2^ in SHIP-1, SHIP-Trend, and Health2006, respectively). Visceral and subcutaneous adipose tissue as well as the common adiposity measures were inversely associated with serum 25OHD concentrations in linear regression models adjusted for age, sex, alcohol consumption, physical activity, smoking status, and month of blood sampling.

**Conclusions:**

Next to common adiposity measures, also abdominal visceral or subcutaneous adipose tissue are inversely associated with serum 25OHD concentrations in the general adult population.

## Background

The classical function of vitamin D in human metabolism is to regulate calcium absorption in the duodenum and reabsorption in the kidney [1]. Vitamin D is consumed either through diet or produced directly in the skin after exposure to sunlight [2]. It is fat-soluble and adipose tissue is its major storage site [2, 3]. In the last years, several studies, e.g. [4–6], demonstrated that obese individuals have lower circulating vitamin D concentrations than non-obese individuals. A meta-analysis [7] confirmed these findings; there was a statistically significant inverse association between the body mass index (BMI) and serum 25-hydroxy vitamin D3 (25OHD) concentrations. Recently, a study using a Mendelian randomization approach suggested a causal relation between BMI and serum 25OHD concentrations [8]. In that study [8], twelve BMI-related single nucleotide polymorphisms (SNPs) and four 25OHD-related SNPs (two related to 25OHD synthesis and two related to 25OHD metabolism) were analysed. It was demonstrated that an increase in BMI leads to a linear reduction in serum 25OHD concentrations. On the other hand, there was no evidence for a regulation of BMI by 25OHD concentrations [8]. The mechanism underlying this observation is not fully investigated yet. One possible explanation for the observed causal relation might be that vitamin D bioavailability is reduced in obesity due to an increased uptake in adipose tissue [9].

In clinical practice, the easiest and most convenient way to assess obesity and overweight is to determine BMI and waist circumference. Yet, these measures contain only limited information regarding fat distribution. This is of importance, as visceral adipose tissue (VAT) better reflects metabolic and cardiovascular risk than subcutaneous adipose tissue (SAT) [10, 11]. Up to now, inverse associations between VAT or SAT and plasma 25OHD concentrations have been reported from a family study including Hispanic and African-Americans [12] and from Canadian residents with European or South Asian ancestry [13]. Moreover, inverse associations between VAT or SAT and serum 25OHD concentrations have been reported from the Framingham Heart Study [14], including 1882 Third Generation participants. While these three studies [12–14] investigated CT-based VAT or SAT, data on MRI-based SAT or VAT is sparse. Moreover, most previous studies assessing the association between common adiposity measures as BMI [5–7, 15], waist circumference [6, 15], waist-to-hip ratio [15, 16], waist-to-height ratio [17], body fat [18, 19] or body surface area [20], focus on the association between one or two selected adiposity measures. In the present study we aimed to assess a comprehensive panel of eight adiposity measures with serum 25OHD concentrations from three cohorts of two large epidemiological studies. These adiposity measures include abdominal VAT or SAT, body fat percentage (%bodyfat), BMI, waist circumference, waist-to-hip ratio, waist-to-height ratio and the body surface area.

## Methods

### Study populations

Data was obtained from the Study of Health in Pomerania (SHIP-1 and SHIP-Trend, conducted in the northeast of Germany) and Health2006 (conducted in Denmark). All investigations were carried out in accordance with the Declaration of Helsinki, including written informed consent of all participants. The survey and study methods of both studies were approved by institutional review boards [SHIP-1 (III UV 73/01) and SHIP-Trend (BB 39/09): ethics committee of the University of Greifswald; Health2006: Ethical Committee of Copenhagen County (KA-20060011) and the Danish Data Protection Agency]. In addition, Health2006 was registered at www.clinicaltrials.gov (Unique ID: KA20060011).

### SHIP-1 and SHIP-Trend

SHIP-1 is the first five-year follow-up of the population-based SHIP-0 cohort. In the baseline examination between 1997 and 2001, a total of 4308 men and women between 20 and 79 years of age participated (response 68.8 %). Out of the 4308 participants, 3300 were re-examined in SHIP-1 between 2002 and 2006. SHIP-Trend is a second population-based cohort in the same study region. In the SHIP-Trend baseline examination between 2008 and 2012, a total of 4420 men and women between 20 and 79 years of age participated (response 50.1 %). Details on sampling methods and study protocols have been reported previously [21, 22]. 25OHD concentrations were measured in all SHIP-1 and a subsample of the SHIP-Trend participants. The SHIP-Trend subsample consisted of the first 1000 study participants without diabetes mellitus. It is thus not truly representative for the whole study population as the subjects included are on average healthier than the whole study population.

#### Interview and physical examination

All participants underwent standardized medical examinations, blood sampling, and an extensive computer-aided personal interview. Data on socio-demographic characteristics and medical histories were collected. Intake of medication was recorded and classified using the anatomical therapeutic chemical classification system (ATC). Participants were defined as physically active, if they reported any physical activity (SHIP-1) or more than one hour of physical activity during summer and winter (SHIP-Trend). During the physical examination, standardized measurements of body height and weight were performed with calibrated scales. Waist circumference and hip circumference were measured using an inelastic tape with the subject standing comfortably with weight distributed evenly on both feet. Waist circumference was measured midway between the lower rib margin and the iliac crest in the horizontal plane. Hip circumference was determined as the greatest circumference between the highest point of the iliac crest and the crotch. BMI was calculated as weight (kg)/height^2^ (m^2^). Waist-to-hip ratio and waist-to-height ratio were calculated from the respective measures. Body surface area was calculated according to the formula of DuBois [23]. Additionally, all SHIP-Trend participants were offered a body impedance analysis (BIA). BIA analyses were performed using a Nutriguard M device and NutriPlus software (Data Input GmbH, Darmstadt, Germany). Charges of 5 kHz, 50 kHz, and 100 kHz were applied to measure resistance, reactance and phase angle. Body fat was automatically calculated from the latter measurements within the NutriPlus software. Abdominal VAT and SAT were measured in SHIP-Trend by magnetic resonance imaging (MRI). The MRI was performed on a 1.5-Tesla system (Magnetom Avanto, Siemens Healthcare AG, Erlangen, Germany, software version syngo MR B15), using a body phased-array coil. For the determination of abdominal fat, axial 3D datasets using the 2-point Dixon technique were acquired (matrix: 256 × 176; slice thickness 4 mm/4 mm/3 mm without gap; 3 × 64 slices; in-phase: TE 4.76 ms, TR 7.48 ms; opp-phase: TE 2.38 ms, TR 7.48 ms). The quantification of abdominal VAT and SAT was done using ATLAS (automatic tissue and labeling analysis software), an in-house developed software at the University of Ulm. The software first performs a fully-automated step [24] followed by a manual correction of the results. The manual correction, performed by certified medical students, included setting the upper (left diaphragm) and lower border (bladder) for the abdominal fat analysis, correcting misclassified fat labels and removing fat labels that do not belong to the abdomen (i.e. arms, breast fat and parenchyma, bone marrow of pelvis and spine).

#### Laboratory measurement

Blood samples were taken from the cubital vein of participants in the supine position. In SHIP-1, participants were mostly non-fasting and blood sampling was performed throughout the day. In SHIP-Trend, participants were fasting and blood sampling was performed between 7.30 a.m. and 1.00 p.m.. Serum 25OHD and serum PTH concentrations were measured on the IDS-iSYS Multi-Discipline Automated Analyser (Immunodiagnostic Systems Limited, Frankfurt am Main, Germany). Serum 25OHD concentrations were measured with the IDS-iSYS 25-Hydroxy Vitamin D assay. The limits of detection and quantitiation were 3.6 and 5.5 ng/ml, respectively. For each analyte three concentrations of control material were measured. In SHIP-1, the coefficients of variation were 16.8 % at low, 13.9 % at medium, and 12.0 % at high concentrations of control material. In SHIP-Trend, the coefficients of variation were 11.6 % at low, 9.1 % at medium, and 10.6 % at high concentrations of control material. Serum creatinine levels were determined with a modified kinetic Jaffé method (SHIP-1: Siemens Dimension RxL; Siemens Healthcare Diagnostics, Eschborn, Germany; SHIP-Trend: Dade Behring Inc, Newark, USA). The eGFR was calculated according to the Cockcroft-Gault formula [25].

### Health2006

The Health2006 study is a population-based cross-sectional study conducted at the Research Centre for Prevention and Health (RCPH). The methodology of the study design has been described previously [26]. Study participants were recruited between June 2006 and May 2008 through the Danish Civil Registration office as a random sample of men and women aged 18–69 years living in the western area of the Capital Region of Denmark. Of 7770 persons invited 3471 participated corresponding to a participation proportion of 44.7 %.

#### Interview and physical examination

Health2006 participants underwent standardized medical examinations, blood sampling, and answered an extensive questionnaire on lifestyle factors and general health. Participants were defined as physically active, if they reported regular sport and exercise or heavy gardening at least three times a week or athletic training. Height and weight were measured wearing light clothes and no shoes. Waist circumference was measured directly on the body surface midway between the lower rib margin and the iliac crest. The hip circumference was measured over light clothing at the widest girth of the hip. BMI was calculated as weight (kg)/height^2^ (m^2^). Waist-to-hip ratio and waist-to-height ratio were calculated from the respective measures. Body surface area was calculated according to the formula of DuBois [23]. Additionally, all participants underwent measurement of impedance for determination of %bodyfat and intra-peritoneal fatness (assessed by ultrasound). Ultrasound measures were performed with the participants lying on their back. VAT was the distance in cm to two decimals between the posterior edge of the abdominal muscles and the front of the lumbar spine. SAT was the distance in cm to two decimals between the front edge of the abdominal muscles and the skin. All ultrasound measures were made after a quiet expiration as described by Stolk et al. [27] with Aquila Pie Medical (Esaote Europe, Maastricht, The Netherlands). The ultrasound measures have been validated against CT/MRI [28]. Fat percentage was measured using a foot-to-foot Tanita Body Composition Analyzer (TBF-300, TANITA Corporation of America, Inc., IL, USA).

#### Laboratory measurement

Fasting venous blood samples from all participants were taken from the cubital vein of participants in the supine position. Serum 25OHD and serum PTH concentrations were measured on the Cobas e411 (Roche Diagnostics GmbH, Mannheim, Germany). Serum 25OHD concentrations were measured with the Vitamin D3 (OH) assay. The limit of detection was 4.0 ng/ml. For each analyte three concentrations of control material were measured. The coefficients of variation were 18.3 % at low, 12.3 % at medium, and 12.0 % at high concentrations of control material. Serum creatinine levels were determined with a modified kinetic Jaffé method (Siemens Healthcare Diagnostics, Eschborn, Germany). The eGFR was calculated according to the Cockcroft-Gault formula [25].

### Exclusions

For the present analyses all subjects with missing information on serum 25OHD concentration (SHIP-1: *n* = 63; SHIP-Trend: *n* = 3425; Health2006: *n* = 62), on adiposity measures (SHIP-1: *n* = 10; SHIP-Trend: *n* = 133; Health2006: *n* = 56) or confounders (SHIP-1: *n* = 12; SHIP-Trend: *n* = 7; Health2006: *n* = 131) were excluded. Moreover, we excluded all SHIP participants, who reported intake of prescribed vitamin D preparations or parathyroid hormone (SHIP-1: *n* = 25; SHIP-Trend: *n* = 7; in Health2006 this information was unavailable), all subjects with suspected hyperparathyroidism, defined as PTH >120 pg/ml, or missing PTH concentrations (SHIP-1: *n* = 29; SHIP-Trend: *n* = 7; Health2006: *n* = 4), with a history of liver disease, extreme alcohol consumption >400 g/day, or missing information on liver disease or alcohol consumption (SHIP-1: *n* = 67; SHIP-Trend: *n* = 38; Health2006: *n* = 8), with renal insufficiency, defined as eGFR <30 ml/min or missing creatinine concentrations (SHIP-1: *n* = 10; SHIP-Trend: *n* = 0; Health2006: *n* = 15), and all pregnant women (SHIP-1: *n* = 12; SHIP-Trend: *n* = 0; Health2006: *n* = 0). This resulted in study populations of 3072 SHIP-1, 803 SHIP-Trend, and 3195 Health2006 participants.

### Statistical analyses

As 25OHD concentrations were measured with different laboratory methods in SHIP and Health2006, the analyses were performed separately for each study. The associations between abdominal VAT, SAT or %bodyfat and 25OHD concentrations were assessed in SHIP-Trend and Health2006. The associations between BMI, waist circumference, waist-to-hip ratio, waist-to-height ratio, or body surface area and 25OHD concentrations were assessed in SHIP-1 and Health2006. Continuous data are expressed as median (1^st^-3^rd^ quartile), nominal data as percentage.

Multivariable linear regression analyses were performed to assess the associations of the adiposity measures (exposure) with serum 25OHD concentration (outcome). The adiposity measures entered the models either categorized in sex-specific quintiles or as continuous variables. We report adjusted mean 25OHD concentrations with 95 % confidence intervals according to quintiles of adiposity measures as well as ß-coefficients with standard errors and *p*-values for a one unit increase in the adiposity measures. As the association between %bodyfat and serum 25OHD concentration appeared to be non-linear in Health2006, we included %bodyfat as linear and as quadratic term in the regression model. To address confounding, we adjusted all models for sex, age (years), alcohol consumption (g/day), physical activity (yes/no), smoking status (non smoker, ex-smoker, occasional smoker, regular smoker), and month of blood sampling. *P*-values <0.05 were considered statistically significant. All statistical analyses were performed with SAS 9.1 (SAS Institute Inc., Cary, NC, USA).

## Results

In SHIP-Trend and Health2006 subjects were on average 50 years of age, while subjects in SHIP-1 were older with a median age of 54 years (Table 1). Women were slightly overrepresented in all three study populations. Population-based differences in lifestyle and health-related behaviour were observed between northeast Germany and Denmark. Danish adults drank on average more alcohol per day and had a higher proportion of risky alcohol consumption. In contrast, German adults had a higher BMI and were more often obese than Danish adults. The median %bodyfat was barley below 30 % in SHIP-Trend and Health2006. SHIP-Trend participants had on average 3.1 and 7.0 l, MRI-based abdominal VAT and SAT, respectively. Health2006 participants had on average 2.8 and 5.9 cm abdominal VAT and SAT layers, respectively.

**Table 1:** Characteristics of the study population

Characteristics	SHIP-1 (*N* = 3072)	SHIP-Trend (*N* = 803)	Health2006 (*N* = 3195)
Male, %	48.7	45.2	45.6
Age, years	54.0 (42.0–66.0)	50.0 (40.0–60.0)	50.0 (40.0–60.0)
Smoking, %			
Non smoker	41.6	42.5	42.5
Ex smoker	32.3	36.2	32.2
Occasional smoker	3.5	3.4	3.2
Regular smoker	22.6	17.9	22.1
Physical activity,			
Any physical activity	40.1	-	-
At least 60 min per week during summer and winter	-	51.4	-
Regular exercise or heavy gardening at least 3 times a week or athletic training	-	-	21.4
Risky alcohol consumption, %^a^	8.7	7.0	22.2
Alcohol, g/day	3.9 (1.0–11.8)	4.2 (1.3–10.5)	10.3 (3.4–22.3)
eGFR, ml/min	97.6 (77.6–121.6)	106.1 (86.7–128.8)	97.7 (81.8–116.6)
25OHD, ng/ml	17.5 (12.5–24.5)	24.9 (17.7–32.7)	16.7 (11.8–22.3)
25OHD <20 ng/ml, %	59.8	33.0	66.0
PTH, pg/ml	34.1 (25.7–44.4)	31.2 (23.8–39.4)	39.0 (30.5–36.1)
VAT (MRI), l	-	3.1 (1.6–5.1)	-
VAT (ultrasound), cm	-	-	5.9 (4.6–7.8)
SAT (MRI), l	-	6.9 (5.2–9.4)	-
SAT (ultrasound), cm	-	-	2.8 (2.1–3.7)
Bodyfat, %	-	28.0 (22.8–33.9)	29.1 (22.9–36.1)
BMI, kg/m^2^	27.4 (24.4–30.8)	26.6 (23.8–29.5)	25.2 (22.2–28.1)
BMI <25 kg/m^2^	29.3	35.1	48.2
BMI 25–29 kg/m^2^	40.8	43.0	36.7
BMI 30–39 kg/m^2^	28.1	21.4	14.1
BMI ≥40 kg/m^2^	1.9	0.5	1.0
Waist circumference, cm	92.2 (82.5–102.0)	86.5 (77.6–96.0)	88.0 (78.0–97.0)
Waist-to-hip ratio	0.86 (0.81–0.93)	0.86 (0.80–0.93)	0.87 (0.80–0.94)
Waist-to-height ratio	0.55 (0.49–0.60)	0.51 (0.46–0.56)	0.51 (0.46–0.56)
Body surface area, m^2^	1.89 (1.74–2.04)	1.89 (1.74–2.03)	1.89 (1.73–2.04)

Data are median (1^st^ – 3^rd^ quartile) or proportions

*BMI* body mass index, *eGFR* estimated glomerular filtration rate according to the Cockcroft-Gault formula, *MRI* magnetic resonance imaging, *PTH* parathyroid hormone, *SAT* abdominal subcutaneous adipose tissue, *VAT* visceral adipose tissue, *25OHD* 25-hydroxy vitamin D

^a^defined as ≥30 g/day in men and ≥20 g/day in women

**Fig. 1:**
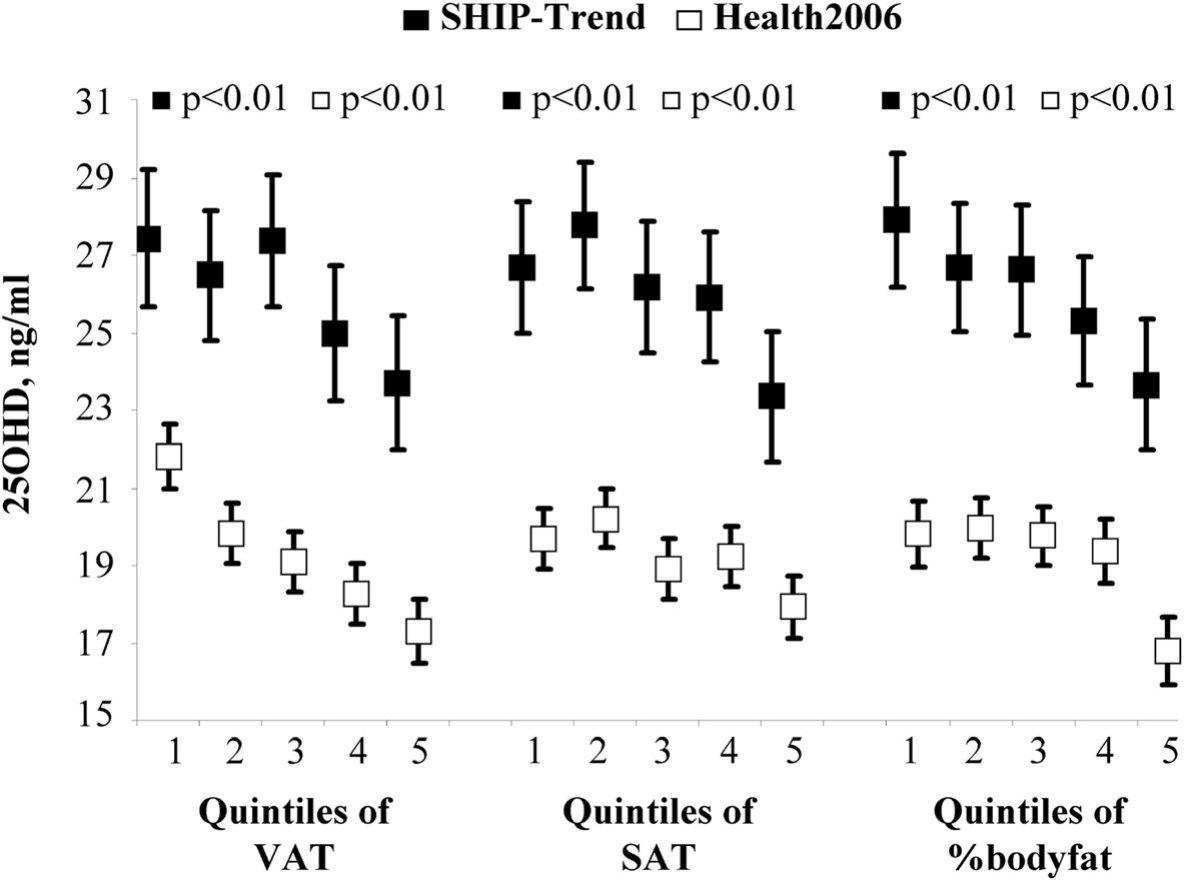
Association between 25-hydroxy vitamin D (25OHD) and adipose tissue. Legend: Adjusted mean serum 25-hydroxy vitamin D (25OHD) concentrations with 95 % confidence intervals according to sex-specific quintiles of visceral and subcutaneous adipose tissue (VAT and SAT) measured by MRI in SHIP-Trend and by ultrasound in Health2006 and of %bodyfat in SHIP-Trend (*N* = 803) and Health2006 (*N* = 3195)

**Fig. 2:**
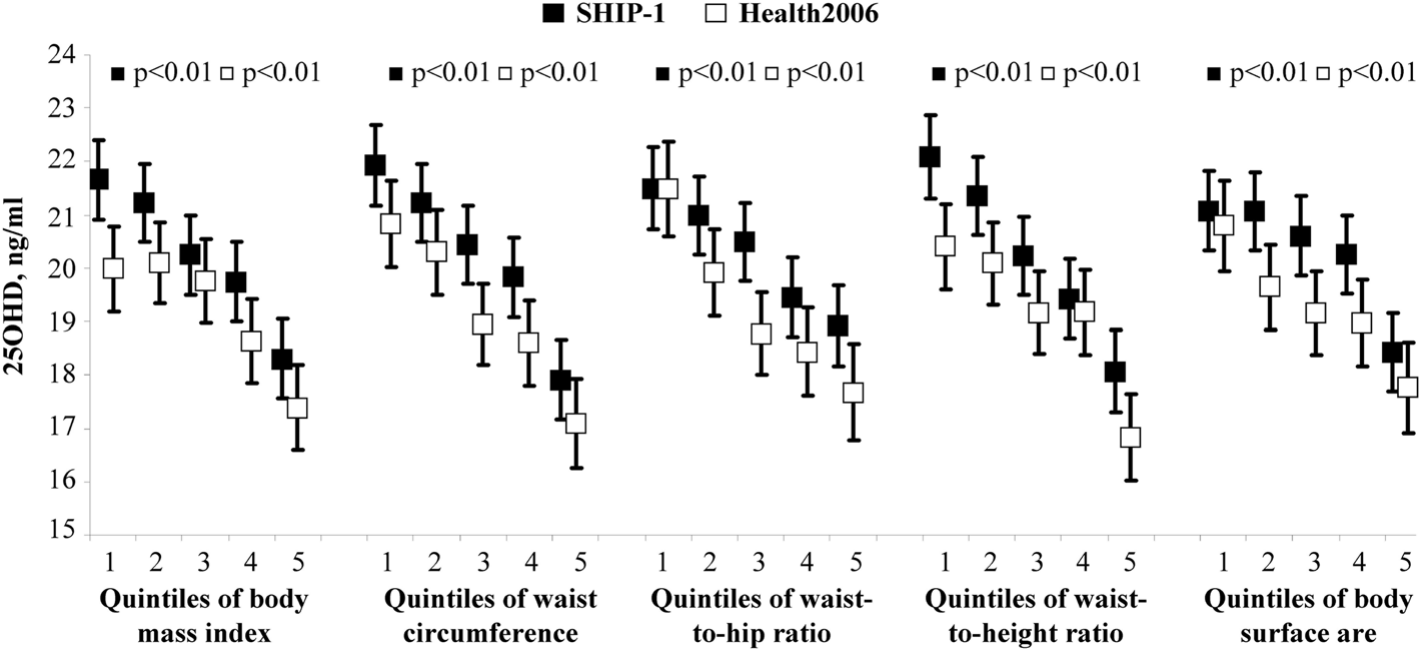
Association between 25-hydroxy vitamin D (25OHD) and standard adiposity measures. Legend: Adjusted mean serum 25-hydroxy vitamin D (25OHD) concentrations with 95 % confidence intervals according to sex-specific quintiles of body mass index, waist circumference, waist-to-hip ratio, waist-to-height ratio and body surface area in SHIP-1 (*N* = 3072) and Health2006 (*N* = 3195)

Adjusted mean 25OHD concentrations decreased across sex-specific quintiles of abdominal VAT or SAT (Fig. 1). Similar to abdominal VAT and SAT, the adjusted mean 25OHD concentration decreased across quintiles of BMI, waist circumference, waist-to-hip ratio, waist-to-height ratio and body surface area (Fig. 2). In fully-adjusted linear regression models the inverse associations between the adiposity measures and serum 25OHD concentration were confirmed (Table 2). For example, we observed that a 1 l increase in VAT was associated with a decrease in the 25OHD concentration of 0.58 ng/ml.

**Table 2:** Associations of different adiposity measures with the serum 25-hydroxy vitamin D concentration

Adiposity measures	Study	ß coefficient	Standard error	*p*
VAT	SHIP-Trend	−0.581	0.174	<0.01
	Health2006	−0.552	0.069	<0.01
SAT	SHIP-Trend	−0.481	0.102	<.001
	Health2006	−0.496	0.117	<0.01
%Bodyfat	SHIP-Trend	−0.266	0.059	<.001
	Health2006	0.469^a^	0.101	<0.01
		−0.010^b^	0.002	<0.01
BMI	SHIP-1	−0.253	0.031	<0.01
	Health2006	−0.250	0.034	<0.01
Waist circumference	SHIP-1	−0.112	0.013	<0.01
	Health2006	−0.112	0.013	<0.01
Waist-to-hip ratio	SHIP-1	−16.212	2.625	<0.01
	Health2006	−14.898	2.370	<0.01
Waist-to-height ratio	SHIP-1	−19.270	2.114	<0.01
	Health2006	−18.837	2.232	<0.01
Body surface area	SHIP-1	−5.090	0.878	<0.01
	Health2006	−5.363	0.883	<0.01

ß-coefficients, standard errors, and *p*-values from fully-adjusted multivariable linear regression analyses. Effect estimates for a one unit increase in the adiposity measures were modeled. For example, in SHIP-1 a one kg/m^2^ increase in BMI is associated with a decrease in the 25OHD concentration of 0.25 ng/ml. All models were adjusted for age, sex, smoking status, physical activity, alcohol consumption, and month of blood sampling

The association between %bodyfat and the 25OHD concentration in Health2006 is non-linear. Therefore, we included %bodyfat and %bodyfat squared in the regression model

*BMI* body mass index, *SAT* abdominal subcutaneous adipose tissue, *VAT* visceral adipose tissue

^a^linear term

^b^squared term

When abdominal VAT and SAT were entered in a single model only SAT remained statistically significantly associated with the 25OHD concentration in SHIP-Trend [ß-coefficient (*p*-value) for SAT: −0.25 (<0.01); ß-coefficient (*p*-value) for VAT: 0.07 (0.77)]. After further adjustment of the model for BMI, SAT still remained significant [ß-coefficient (*p*-value) for SAT: −0.58 (0.01) ß-coefficient (*p*-value) for VAT: 0.05 (0.85)]. Regarding the Health2006 cohort, both VAT and SAT remained significant when entered in a single model [ß-coefficient (*p*-value) for SAT: −0.24 (0.045); ß-coefficient (*p*-value) for VAT: −0.51 (<0.01)]. After further adjustment for BMI, VAT still remained significant [ß-coefficient (*p*-value) for SAT: −0.09 (0.54) ß-coefficient (*p*-value) for VAT: −0.39 (<0.01)].

In addition, there appeared to be a non-linear association between %bodyfat and the 25OHD concentration in Health2006 (Fig. 1). This association was confirmed when %bodyfat entered the model as linear and quadratic term.

## Discussion

The present cross-sectional study revealed inverse associations between abdominal VAT or SAT and serum 25OHD concentrations in general adult populations from northeast Germany and Denmark. Moreover, previously reported inverse associations between common adiposity measures and serum 25OHD concentrations were confirmed.

The inverse associations between the adiposity measures and the 25OHD concentration observed in the three cohorts were alike, and thus strengthen each other. Previous studies using CT-based [13, 14] or MRI-based VAT and SAT [18] reported similar results. For example, in 182 European and 188 South Asian subjects [13], VAT and SAT were associated with 25OHD concentrations in women, while in men only SAT was associated with 25OHD. When VAT, SAT and %bodyfat were included in the same model, VAT was associated with 25OHD concentrations in both sexes [13]. In another study including 1882 Framingham Third Generation participants [14], VAT and SAT were both associated with 25OHD concentrations. After adjustment for waist circumference or when VAT and SAT entered the same model, only VAT remained associated with 25OHD [14]. In 567 Chinese men VAT and SAT correlated significantly with 25OHD concentrations in unadjusted analysis [18]. In multivariable linear regression models adjusted for BMI, VAT but not SAT was significantly inverse associated with 25OHD concentrations [18]. Moreover, in 60 postmenopausal women [29], the prediction of MRI-based VAT improved after including the 25OHD concentration and four further biomarkers in a model consisting of age, ethnicity, BMI, waist circumference, and waist-to-hip ratio [29]. In the present study VAT and SAT were both strongly associated with the 25OHD concentration. When VAT, SAT and BMI were entered in a single model only VAT (Health2006) or SAT (SHIP-Trend) remained significant. Thus, our data provide no final conclusion on whether VAT or SAT is more robustly associated with the 25OHD concentration.

As with VAT and SAT previous studies in adolescents [30], post-menopausal women [19] or men [18], ethnic diverse populations [13], or obese and non-obese women [31–33], demonstrated inverse relations between bodyfat and 25OHD concentrations. These associations were present irrespective if bodyfat was given as proportion [13, 18, 30, 32] or as mass [19, 30, 31, 33, 34] and irrespective if fat mass was determined by dual X-ray absorptiometry [13, 19, 30, 31, 34] or by body impedance [18, 32, 33]. Inverse linear associations of %bodyfat [13, 18], total [34] or total and regional fat mass [19] with 25OHD concentrations were reported from these studies. This is in agreement with our results: subjects with a high %bodyfat have lower 25OHD concentration than subjects with a low %bodyfat.

Moreover, data on the association between total body surface area and 25OHD concentrations is sparse. To the best of our knowledge, only one previous study analyzed this relation in 489 apparently healthy subjects [20]. The authors found a significant positive relationship between height, body surface area and the 25(OH)D concentration. This positive association was explained with increased synthesis of vitamin D in the skin due to increasing body surface area. Thus, taller people may have higher 25OHD concentrations [20]. Concurrently, BMI and waist circumference are highly correlated with body surface area (spearman correlation coefficients in SHIP-1 for BMI: 0.61, *p*-value: <0.01, for waist circumference: 0.75; *p* < 0.01). An increase in BMI or waist circumference may not be related to an increase in skin exposure to UV-B radiation. Thus, the inverse association between body surface area and the 25OHD concentration revealed in the present study also seems to be plausible.

Previously, several mechanisms underlying the association between obesity and vitamin D deficiency have been proposed. One theory suggests a volumetric dilution effect [35]. The lipophilic vitamin D_3_ molecule can be stored in adipose tissue, leading to a reduced serum bioavailability [4, 36]. With increasing fat mass, vitamin D_3_ is distributed in an increasing mass of adipose tissue and thus serum bioavailability is reduced [35, 37]. Indeed, the response to a given UV-B radiation dose is lower in obese compared to non-obese subjects [38]. Moreover, vitamin D catabolism may be increased in obesity, as the 25OHD degrading enzyme CYP24A1 is increased in obese compared to lean subjects [37]. Other possible mechanisms explaining the low serum 25OHD concentrations in obesity include a reduced synthesis of vitamin D3 in the skin, a smaller skin area exposure to sunlight, a lack of outdoor activity [36, 39], and a negative feedback regulation of hepatic 25OHD synthesis exerted by elevated 1,25-dihydroxy vitamin D and parathyroid hormone concentrations [35]. It was thus suggested, that adipose tissue is involved in the regulation of serum 25OHD concentrations [4].

The present study was performed in a large number of intensively characterized adults from the general population in northeast Germany and Denmark. The size of the study populations allowed us to determine robust estimates for the analyzed associations. Next to these strengths our study has several limitations that need to be considered. One limitation is the cross-sectional study design, which prohibits detecting any causality in the investigated associations. Yet, a previous study [8] demonstrated that BMI causally affects the 25OHD concentration. We thus speculate that the anthropometry, as characterized by the different adiposity measures, causally effects the 25OHD concentration. Another limitation is the difference in 25OHD measurement and the distinct VAT or SAT determination (MRI or ultrasound-based), which prevented us from pooling the data. Moreover, SHIP and Health2006 participants taking non-prescribed vitamin D preparations were not excluded from the analyses. If supplement intake varies between obese and non-obese subjects, it might cause an under- or overestimation of the reported effect. Although the intake of non-prescribed vitamin D preparations was highest in the lowest BMI quintile it was equal among the four highest BMI quintiles in Health2006, therefore we assume our results to be unaffected. Further, there is no information on dietary vitamin D intake in the present study. Again, differences between obese and non-obese subjects may have affected our results. A last limitation of our study is that all analyses are based on single-occasion 25OHD measurements, which were taken throughout the year.

## Conclusion

Taken together, our study suggests that next to standard adiposity measures, also MRI or ultrasound-based abdominal VAT or SAT are inversely associated with the 25OHD concentration. The associations between the adiposity measures and the 25OHD concentration were strikingly similar between northeast Germany and Denmark despite differences in lifestyle and despite different methods of adipose tissue quantification.
